# Transoral robotic resection of large intramural, cervical esophageal lipoma—Report of a case and description of technique

**DOI:** 10.1002/ccr3.7009

**Published:** 2023-03-02

**Authors:** Bharat Akhanda Panuganti, Harishanker Jeyarajan

**Affiliations:** ^1^ Department of Otolaryngology‐Head and Neck Surgery The University of Alabama at Birmingham Birmingham Alabama USA

**Keywords:** dysphagia, esophageal lipoma, minimally invasive surgery, transoral robotic surgery

## Abstract

Transoral robotic surgery (TORS) has evolved into a common surgical modality used to treat primarily oropharyngeal malignant and benign pathologies. The single port Intuitive Surgical da Vinci surgical robotics system facilitates access to the hypopharynx and cervical esophagus. We aim to describe our approach and advantages of the technique.

## Graphical abstract

Transoral robotic surgery should be considered as a surgical approach for hypopharyngeal and cervical esophageal masses. The da Vinci single‐port (SP) robotic system conveys significant advantages in surgical dissection, exposure, and visualization.

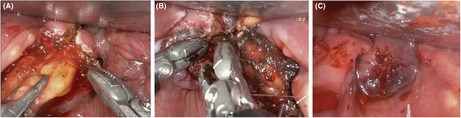

## 1 INTRODUCTION

Transoral robotic surgery (TORS) is a common, minimally invasive surgical approach to treat benign and malignant head and neck pathology. The ability to expose and operate on pharyngeal pathology robotically has often mitigated the need for open, more morbid approaches. Development in the field of surgical robotics has expanded the geography of head and neck pathology that is routinely accessible, particularly since the evolution of the da Vinci single‐port (SP) robotic system (Intuitive Surgical). A recent study, for example, found that robotic surgical resection, versus transoral laser microsurgical resection, of T1/T2 hypopharyngeal cancer was associated with improved overall survival in the setting of a significantly lower rate of positive surgical margins. This was theorized to be related to dynamic tumor visualization and improved surgical ergonomics.^1^ In spite of this emerging data, robotic surgery in the hypopharynx and cervical esophagus (TORS‐HE) is considered less frequently than in the oropharynx. We present for the first time in the extant literature a TORS resection of a post‐cricoid and cervical esophageal lipoma. The authors of this study aimed to report on the feasibility of cervical esophageal transoral robotic surgery, so that surgeons might more frequently consider the approach among patients with permitting anatomy and pathology.

## 2 REPORT OF A CASE

**FIGURE 1:**
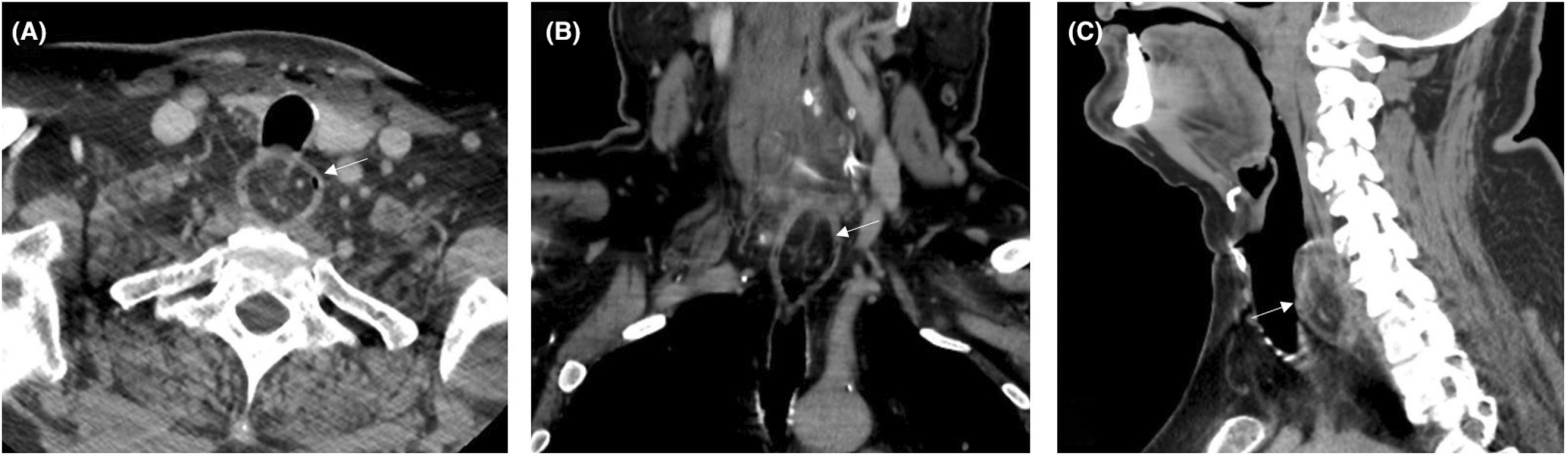
Computer tomography scan demonstrating a fatty mass (2.4 × 2.6 × 3.8 cm) extending from the level of the cricoid cranially into the cervical esophageal wall caudally. Location of the lipoma indicated by the white arrow.

We present a 74‐year‐old male patient who initially arrived in the head and neck surgery clinic with dysphagia to solid foodstuff, the sensation of food‐sticking, and imaging demonstrating a large upper esophageal lipoma. He previously underwent a debulking procedure via rigid esophagoscopy with temporary improvement in his symptoms approximately 2 years prior to his contemporary presentation; pathology demonstrated a benign lipoma. A preoperative barium esophagram demonstrated mild hypopharyngeal stasis and evidence of lower esophageal sphincter (LES) dysfunction. Computed tomography (Figure 1A/B/C) demonstrated a large fatty mass extending from the level of the cricoid cranially into the cervical esophagus caudally (2.4 × 2.6 × 3.8 cm), with distal esophageal dilatation. The mass was well‐circumscribed, without calcifications or evidence of transmural infiltration. In‐office flexible laryngopharyngoscopy revealed mobile arytenoids, a clear hypopharynx without retained secretions, and bulging of the post‐cricoid mucosa with submucosal nodularity. Given that his primary complaint was food‐sticking in his throat, we elected to proceed initially with management of the lipoma. He was counseled regarding the option of a standard transoral, microscopic approach to the lipoma using a pharyngoscope, but was given the alternative option of a transoral robotic approach using the daVinci single‐port (SP) robot (Intuitive Surgical) and Olympus Feyh–Kastenbauer (FK‐WO) transoral retractor system. The authors hypothesized that a robotic exposure might improve visualization using the fully articulating SP endoscope, and optimize both the proximal and the distal working space, unrestricted by the confines of a tubular scope.

### 2.1 Description of surgery

**FIGURE 2:**
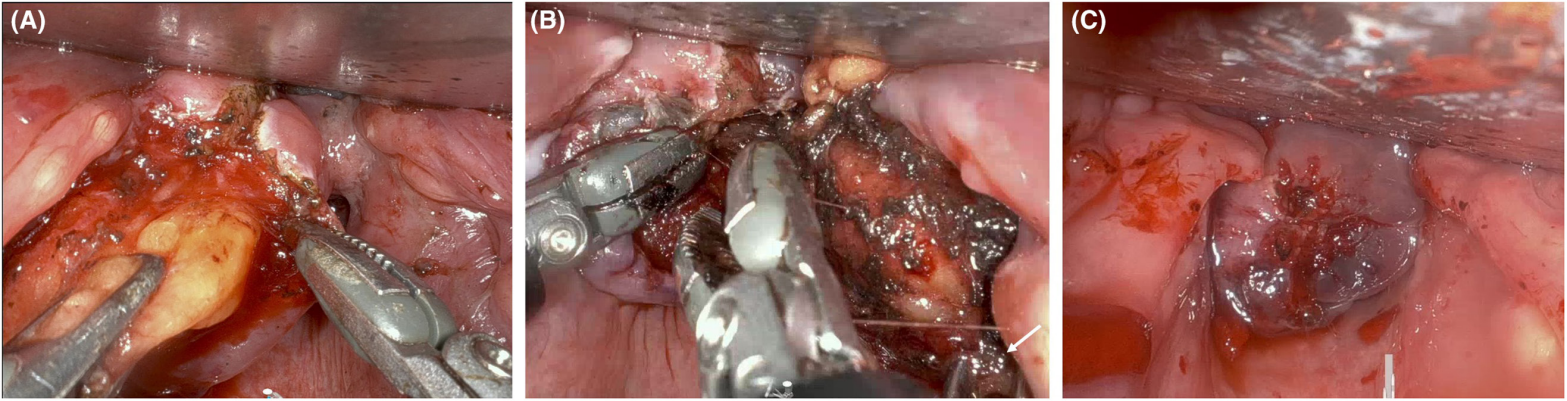
Intraoperative stills obtained at different portions of the surgery. (A) The mass is dissected free of the adjacent post‐cricoid mucosa and splayed cricopharyngeal muscular fibers. (B) Maryland bipolar forceps are used to retract the lipoma laterally (indicated by white arrow), while two other instruments are used to continue dissecting the mass from the adjacent mucosa. (C) The wound is closed primarily with 3‐0 Monocryl barbed suture.

**FIGURE 3:**
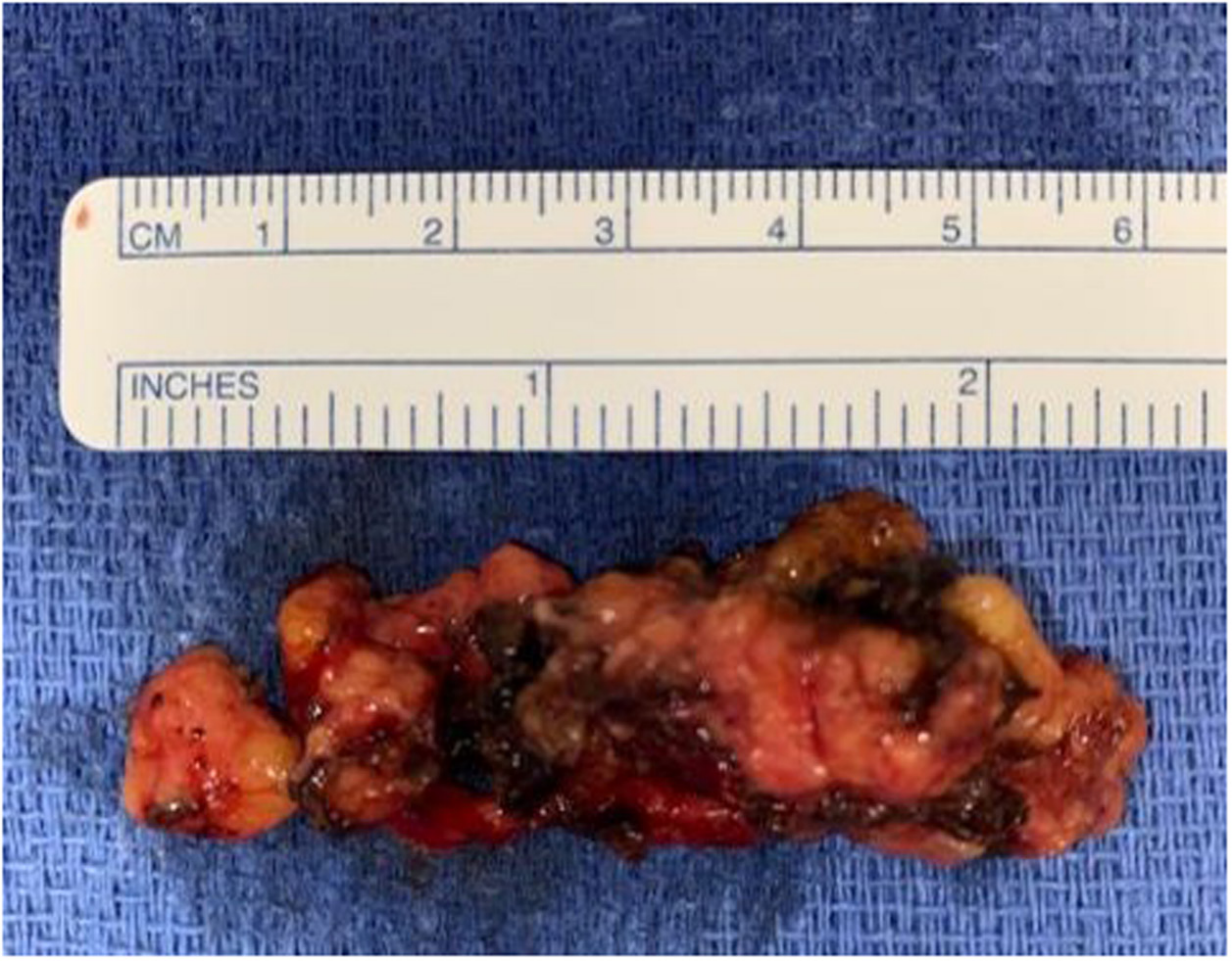
Ex vivo still of the lipoma, resected nearly completely en bloc.

The patient underwent general endotracheal anesthesia with a 6‐0 reinforced, laser‐safe endotracheal tube. He was placed into suspension with a curved tongue blade with the distal tip situated posterior to the larynx using the Olympus FK‐WO retractor system. With the larynx suspended anteriorly, the lipoma was readily apparent. The da Vinci SP robot was brought into dock, and the procedure began with a carbon dioxide‐laser assisted incision. A flexible laser fiber fit with a red rubber catheter was passed through the facet engaged with the Maryland retractor. A ~2.5 cm incision in the post‐cricoid mucosa was made with the laser, and fatty lobules of the lipoma immediately became apparent. A combination of blunt dissection and bipolar cautery was performed, separating lipoma from the adjacent cricopharyngeal muscle fibers and post‐cricoid and proximal esophageal mucosa (Figure 2A). All three robotic arms were deployed, wherein one Maryland bipolar forceps was used to retract mass/mucosa, and two other instruments were used to perform the dissection (Figure 2B). Most of the fatty mass was confluent and removed en bloc (Figure 3). A hemostatic agent was placed in the wound bed, and the vertical mucosal defect closed using 3–0 barbed Monocryl suture in simple running fashion (Figure 2C). A post‐operative day 1 barium esophagram demonstrated an integral closure with no extraluminal leakage. He resumed a regular diet with no precautions and reported improvement in his dysphagia on post‐operative follow‐up.

## 3 DISCUSSION

We describe for the first time in the literature a TORS resection of a cervical esophageal mass. Although upper esophageal lipomas are a relatively rare clinical entity (0.4% of all benign gastrointestinal lesions, with vast majority of esophageal lipomas [88.6%] manifesting as intraluminal stalked lesions), the authors of this study primarily aimed to establish the feasibility of transoral robotic cervical esophageal surgery with the report of this case.^2^

A common concern with TORS is exposure. Cervical lordosis and prior history of radiotherapy have been identified specifically as predictors of difficult hypopharyngeal exposure for transoral robotic surgery,^3^ though any characteristic complicating the displacement of oropharyngeal soft tissues (e.g. large tongue, narrow mandible, compliance of the upper neck soft tissue, and mandibular tori) or the placement of robotic instrumentation (e.g. narrow interincisal distance) should be considered prior to TORS‐HE.^4^ However, these same anatomic constraints apply to standard transoral endoscopic techniques using a laryngoscope or pharyngoscope.^5^

Contrarily, TORS‐HE offers unique technical advantages when operating in the post‐cricoid and upper esophageal region. Using the da Vinci SP system, the authors were able to use three robotic instruments through a single cannula, permitting dynamic retraction of the adjacent, redundant post‐cricoid mucosa with one instrument, and bimanual dissection of the lipoma with the others. The capability of distal, instrumented retraction and the critical advantage of a flexible endoscope (unique to the SP system) help the surgeon overcome the technical challenges of static transoral exposure. The ability to engage three surgical instruments while maintaining a wide‐field, stereoscopic view of the post‐cricoid/cervical esophageal region trans orally is an important technical advantage of robotic, versus the standard, transoral microscopic, exposure.

## 4 CONCLUSION

TORS‐HE should be considered a safe and effective surgical option for cervical esophageal pathology. The da Vinci SP system, allowing for distal instrumented retraction, dynamic visualization with a flexible endoscope, and surgical maneuvering unrestricted by the confines of a tubular scope, constitutes a significant technical advantage in transoral hypopharyngeal and cervical esophageal surgery.

## AUTHOR CONTRIBUTIONS

**Bharat Panuganti:** Conceptualization; data curation; formal analysis; investigation; methodology; writing – original draft; writing – review and editing. **Harishanker Jeyarajan:** Conceptualization; investigation; methodology; writing – review and editing.

## FUNDING INFORMATION

None.

## CONFLICT OF INTEREST STATEMENT

None.

## CONSENT

Written informed consent was obtained from the patient to publish this report in accordance with the journal's patient consent policy.

## Data Availability

None.
